# Novel Imidazole Liquid Crystals; Experimental and Computational Approaches

**DOI:** 10.3390/molecules27144607

**Published:** 2022-07-19

**Authors:** Nada S. Al-Kadhi, Fowzia S. Alamro, Saheed A. Popoola, Sobhi M. Gomha, Noha S. Bedowr, Shahd S. Al-Juhani, Hoda A. Ahmed

**Affiliations:** 1 Department of Chemistry, College of Science, Princess Nourah bint Abdulrahman University, P.O. Box 84428, Riyadh 11671, Saudi Arabia; nsalkadhi@pnu.edu.sa (N.S.A.-K.); fsalamro@pnu.edu.sa (F.S.A.); 2 Chemistry Department, Faculty of Science, Islamic University of Madinah, Madinah 42351, Saudi Arabia; abiodun@iu.edu.sa; 3 Department of Chemistry, Faculty of Science, Cairo University, Cairo 12613, Egypt; 4 Chemistry Department, College of Sciences, Taibah University, Yanbu 30799, Saudi Arabia; nbedowr@taibahu.edu.sa (N.S.B.); tu4053324@taibahu.edu.sa (S.S.A.-J.)

**Keywords:** imidazole liquid crystals, mesomorphic properties, smectic phase, DFT, optimized structures

## Abstract

The liquid crystalline materials named (*E*)-4-(2-(4-oxo-5,5-diphenyl-4,5-dihydro-1H-imidazol-2-yl)hydrazineylidene)methyl)phenyl and 4-(alkoxy)benzoate, **In**, were synthesized and their mesomorphic behaviors were examined. The chemical structures of the produced compounds were confirmed by Fourier-transform infrared spectroscopy (FT-IR), NMR, and elemental analysis. Differential scanning calorimetry (DSC) and polarized optical microscopy were used to investigate the mesomorphic properties of designed heterocyclic derivatives. All the compounds tested had suitable thermal stability and enantiotropic behavior of smectogenic temperature ranges. Furthermore, the enantiotropic smectic C phases were observed to cover all the homologues. Moreover, computational investigations corroborated the experimental findings of the mesomorphic behavior. The reactivity parameters were computed for the derivatives and linked with the experimental data. Theoretical calculations revealed that the polarizability of the studied series increases with the chain length, whereas the HOMO–LUMO energy gap or other reactivity descriptors were less sensitive to the size of the system. On the other hand, the predicted thermodynamic parameters revealed the size dependence of thermal stability of the compounds.

## 1. Introduction

The imidazole ring is a well-known five-membered, nitrogen-containing heterocyclic scaffold. Interestingly, the imidazole-based molecules have emerged as a critical component of pharmaceutical and medical chemistry [1,2,3,4,5,6,7,8]. Imidazole derivatives have a wide range of industrial applications. It is the perfect building block for nonlinear optical materials because of its great electron-withdrawing potential and good coplanarity [9,10,11]. Other uses for imidazolium salts include coating metal nanoparticles to offer antimicrobial action, removing metal ions from aqueous solutions, and producing oriented liquid crystals [12].

Researchers witnessed a tremendous growth in the number of studies undertaken in the field of liquid crystals (LCs) during the 1980s. Imidazole salts are one of the potential liquid crystal compounds. Imidazoles can be quaternized to generate ionic liquids and LCs [13,14,15].

In fact, it can be employed as a stabilizing substance for gold nanoparticles in the manufacturing of dye-sensitized solar cells and light-emitting electrochemical cells [16,17,18]. Furthermore, it has a potential of being used as a conducting material [19].

Many studies have been conducted to investigate the properties of relatively small aromatic compounds, such as imidazolium, pyridinium, and others, that are substituted by one or more aliphatic tails [20].

In recent years, it has been recognized that hydrazones not only serve as valuable synthetic intermediates [21,22,23] with a variety of biological activities [24,25,26], but also find applications in supramolecular switches, metalloassemblies, and sensors [27]. Based on Paulus’s pioneering work on liquid crystalline hydrazones [28], more subsequent works by numerous authors [29,30,31,32,33,34,35] have affirmed the hydrazones as an ideal building blocks for complex functional liquid crystalline materials [36].

Different azomethine/ester homologous series of liquid crystals (LCs) have been thoroughly researched to appreciate the relation between chemical structure and mesomorphic attributes because of their unique mesomorphic properties [37,38,39,40,41]. An organic molecule’s molecular shape generally affects the mesophase stability and temperature ranges it can withstand; even a little alteration in molecular geometry can have a significant impact on mesomorphic behavior [42].Moreover, the Schiff base (azomethine group) serves as a connecting group within the molecule’s rigid core. The azomethine group maintains molecular linearity despite having a stepped core structure, making the molecule more stable and enabling the evolution of mesophase [43,44,45,46,47,48].

**Scheme 1:**
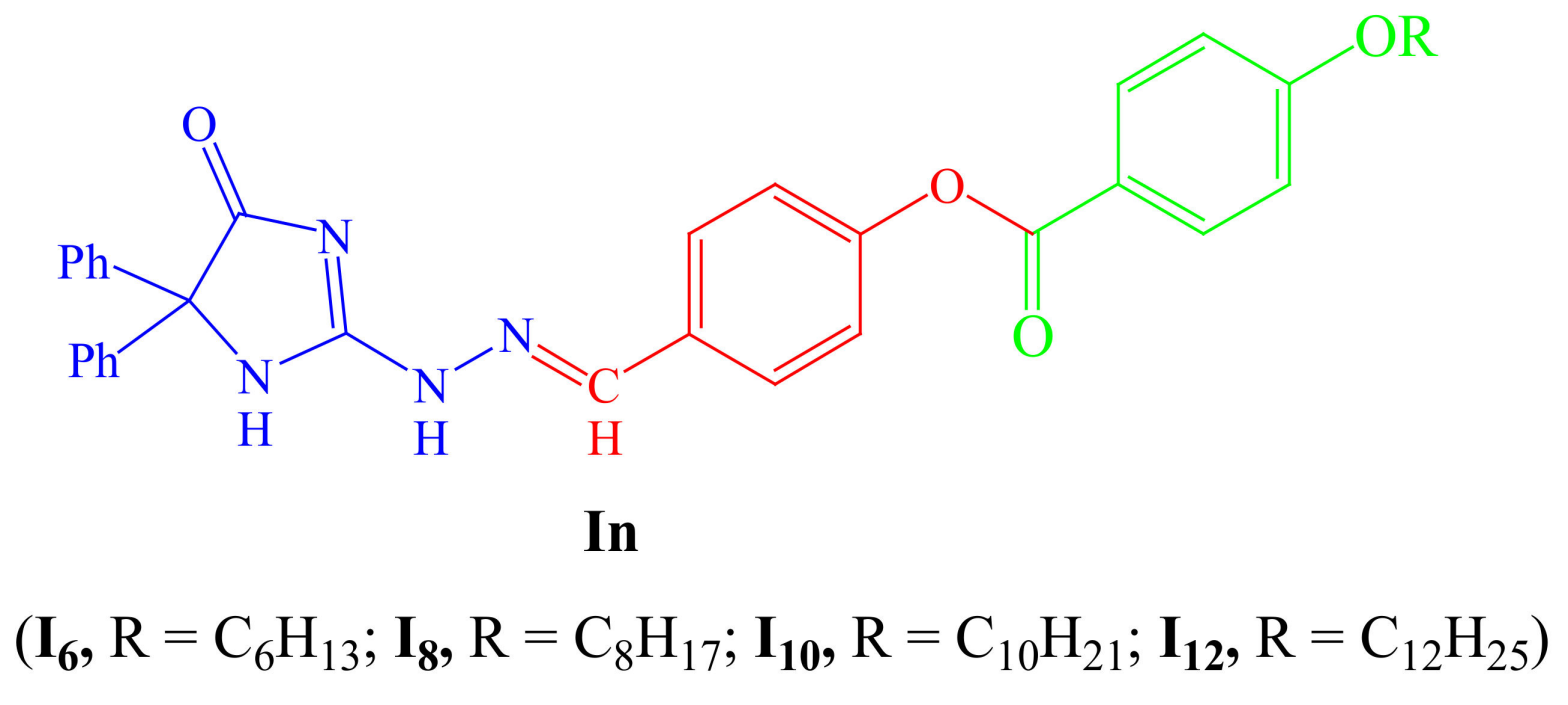
The designed series **In**.

To completely grasp the structure–property relationship, we focus our efforts on the new imidazole hydrazones liquid crystalline derivatives with ester links. In this study, a series of (*E*)-4-(2-(4-oxo-5,5-diphenyl-4,5-dihydro-1H-imidazol-2-yl)hydrazineylidene)methyl)phenyl 4-(alkoxy)benzoate, Scheme 1 (**In**), were created and subjected through theoretical and experimental analysis to determine how the mesogenic core’s structure and the length of the terminal alkoxy chain affect the mesophase behavior of the understudied compounds.

## 2. Results and Discussion

### 2.1. Mesomorphic Studies of Series, **In**

**Figure 1:**
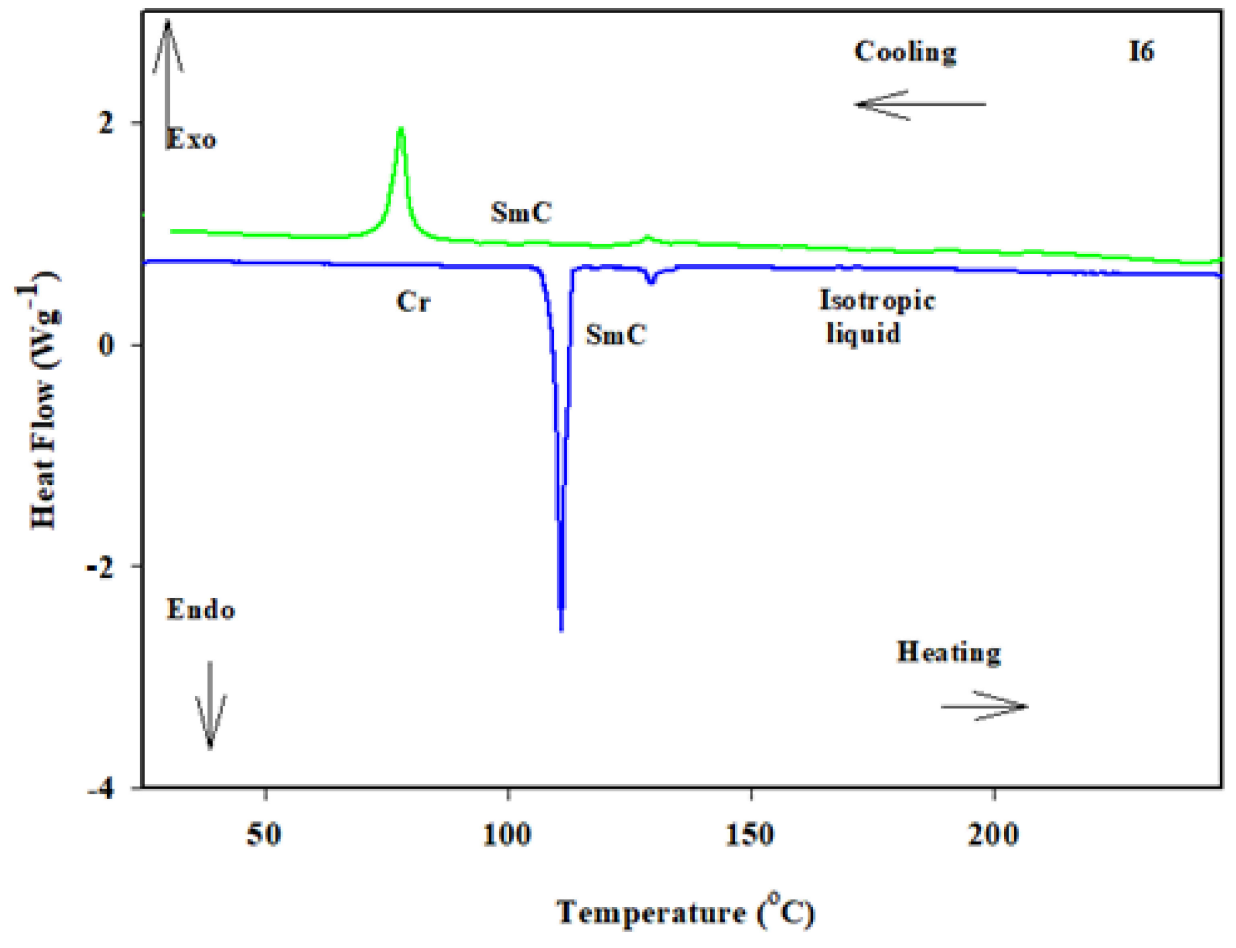
DSC curve of derivative **I6** upon heating/cooling rounds with heating rate 10 °C/min.

**Figure 2:**
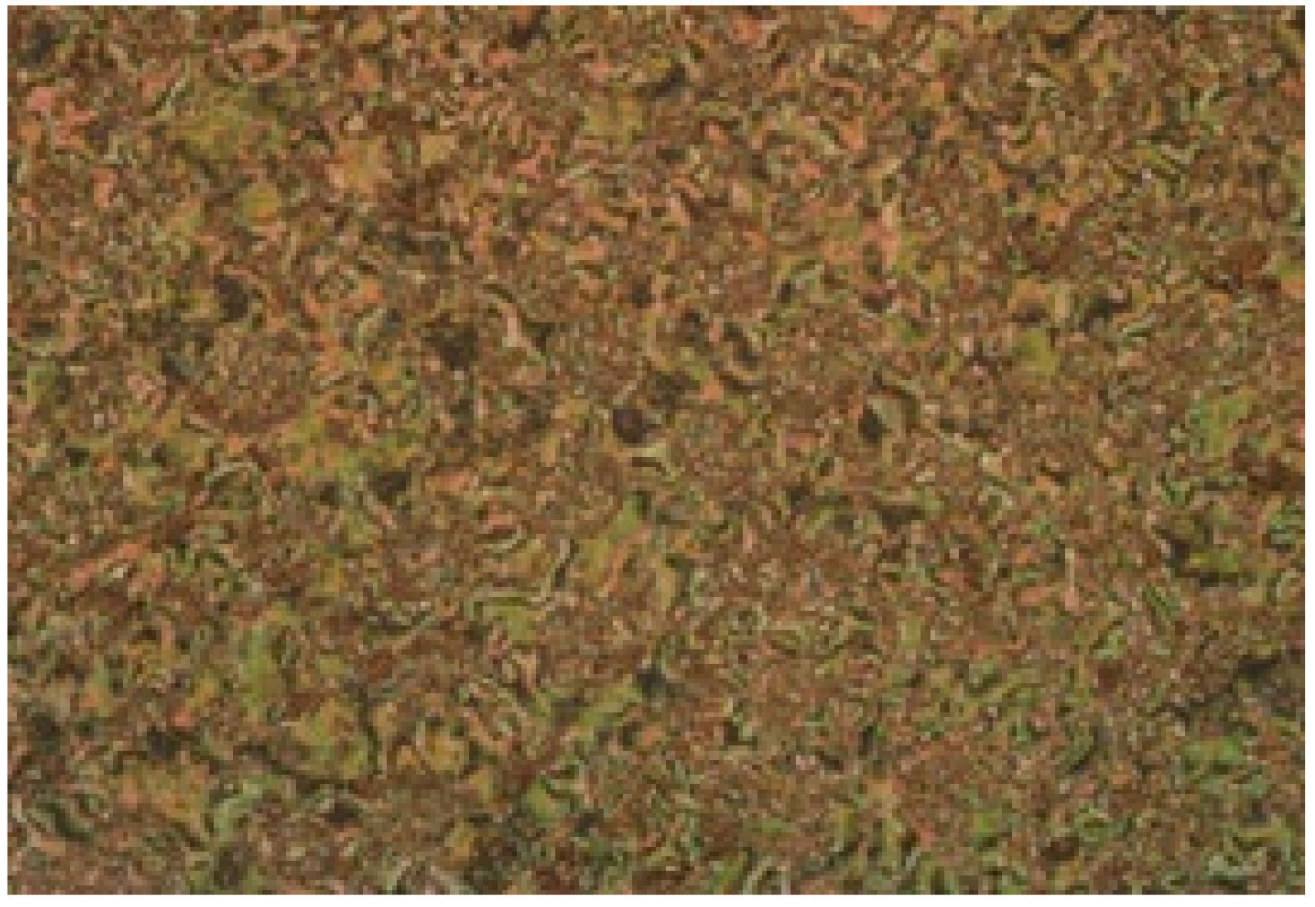
SmC phase texture (50 µm) at 118.0 °C upon heating of sample **I6** under POM.

**Figure 3:**
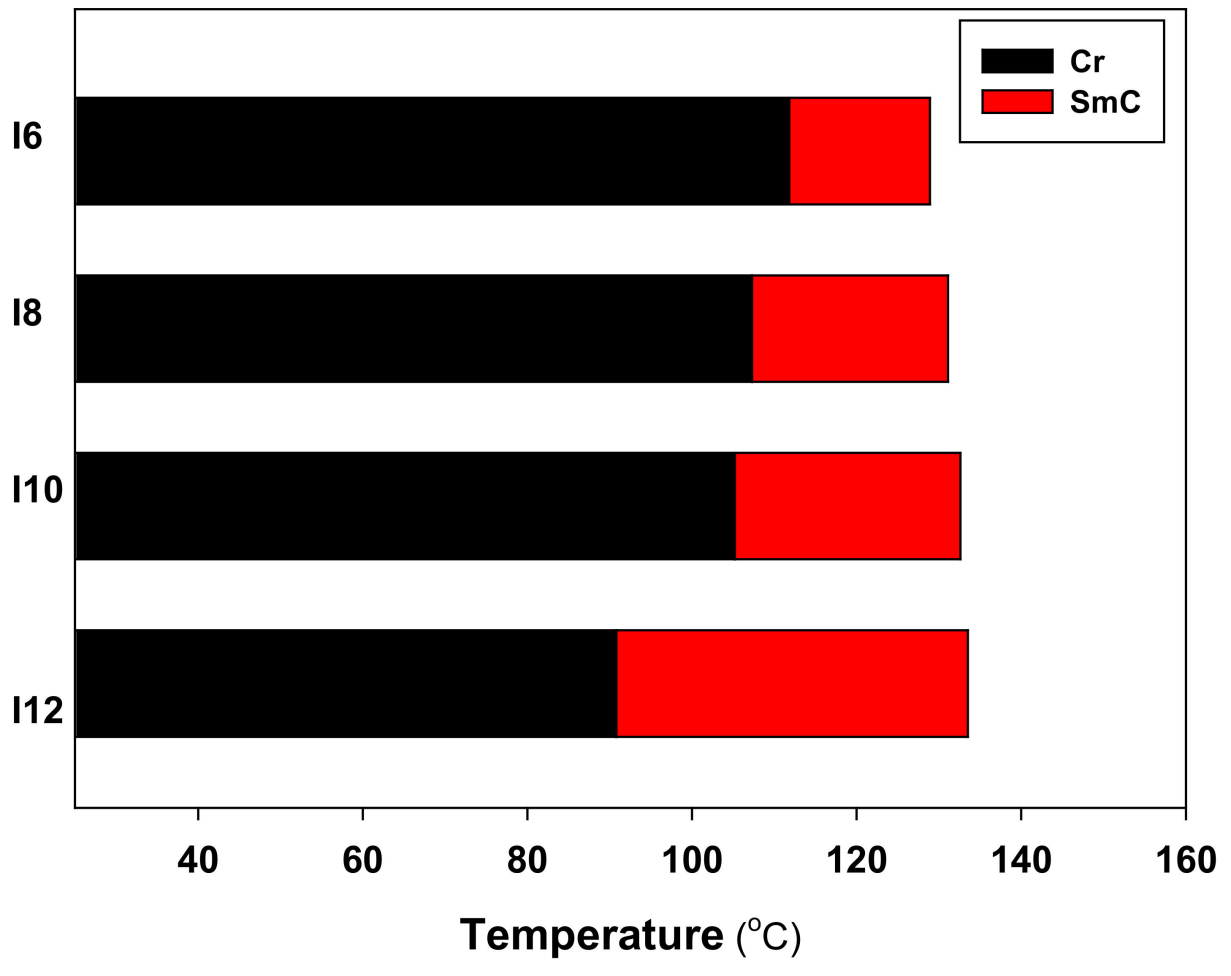
DSC graphical transitions of studied derivatives, **In**.

Investigations were made into the mesomorphic characteristics of the current synthetic series (**In**). The findings of DSC measurements of transition temperatures and enthalpies are presented in Table 1. To check for compound stability, DSC values from the second heating/cooling cycle were measured. The thermal properties of all of these compounds were recorded using the second heating scan. Figure 1 shows the DSC curve of the synthesized homologue **I6** after heating and cooling scans. The heating of the homologue produced two endothermic peaks, which were assigned to the crystal-to-smectic and smectic-to-isotropic transitions, while the cooling produced two exothermic peaks, as illustrated in Figure 1. The DSC data were corroborated by the POM textures. The POM textures supported the DSC data. The POM exhibited smectic C (SmC) phase textures (Figure 2). All homologues have enantiotropic monomorphic characteristics, as illustrated in Figure 1 and Figure 2. Figure 3 displays a graphic representation of DSC transition temperatures to assess how the terminal flexible alkoxy chain affects the mesophase behavior of the investigated derivatives (**In**). As demonstrated in Table 1 and Figure 3, all members of the current series (**In**) are enantiotropic, have acceptable thermal stabilities, and have mesomorphic temperature ranges. The mesomorphic behavior of any proposed liquid crystalline architecture is generally determined by the type of bridging groups and the length of terminal chains [49,50]. The melting points (Cr–to–SmC) follow a regular pattern, as illustrated in Table 1 and Figure 3. The homologue **I12** has the lowest melting point (Cr-SmC = 90.8 °C), while the homologue **I6** has the highest melting point (Cr-SmC = 111.8 °C). The synthesized group exhibits purely SmC mesophases and the SmC phase stability increase with n, as previously described [51,52,53].

In general, the most important factor affecting mesophase behavior is the polarity and/or polarizability of molecules’ mesogenic cores. Table 1 and Figure 3 show that the investigated mesomorphic temperature range of **In** series (**Δ*T***_SmC_) rises with n. In comparison to the other members, homologue **I12** has the largest smectogenic temperature range and thermal stability, while homologue **I6** has the smallest SmC temperature range and thermal stability. According to the current findings, the stability of the resultant mesophases rises when molecular anisotropy increases owing to a change in the mesogenic core of the molecule. The type, stability, and temperature range of the produced mesophases, on the other hand, are influenced by the length of the terminal group. In a nutshell, the derivatives’ geometrical features allow for the development of SmC mesophases. As is commonly recognized, many features of rod-like molecules influence their mesomorphic characteristics, including polarizability, dipole moment, aspect ratio, and competitive contact between terminal moieties. Thus, the mesomeric configurations affect molecular geometry, which in return affects molecular–molecular interactions. Our studies showed that molecular aggregation between calamitic molecules, caused by the lateral attraction of planar molecules enforced by longer alkoxy-chains, was having an impact on the thermal stabilities of the mesophases (n). Rod-shaped LC molecules stacked up because to molecular aromatic molecular π–π stacking attraction between co-planar molecules. However, these interactions were enhanced by an increase in alkoxy-chains (n). Another element that changes depending on mesomeric effects is the end-to-end contact of terminal flexible chains. These characteristics alter the mesomorphic properties of components in various ratios.

The transition normalized entropy changes (**Δ*S***_SmC-I_/R) for series **In** were calculated and are shown in Table 1. The entropy changes were found to have an asymmetric relationship with the terminal alkoxy chain length, n. The observed little entropy shifts in the examined compounds could be attributed to appropriate weak conjugative interactions between the mesogenic cores connecting the opposite to each other at the molecule’s central imidazolone group [54,55].

### 2.2. Theoretical DFT Studies

#### Reactivity Parameters

**Figure 4:**
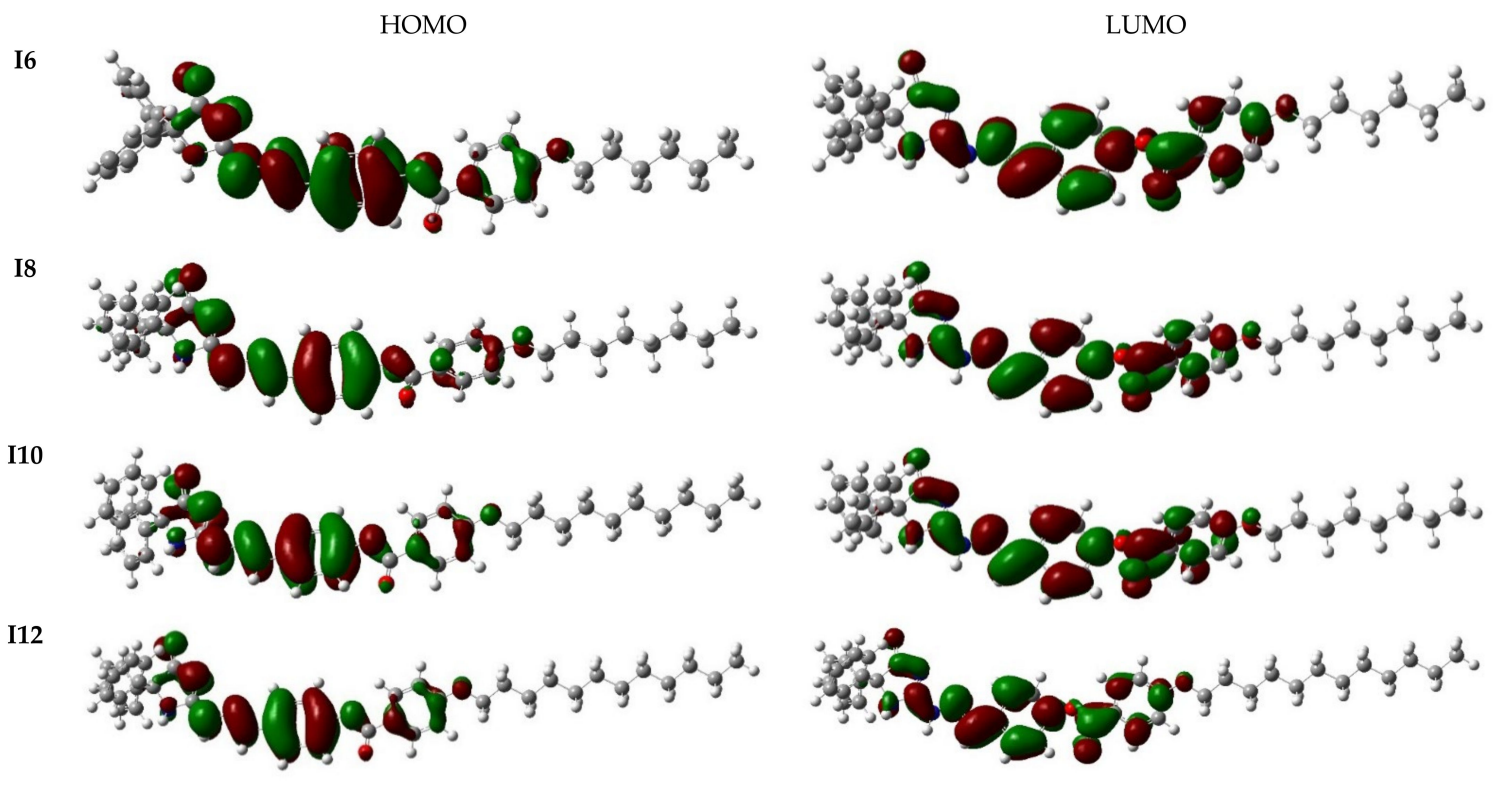
FMO’s computed at B3LYP/6-31g(d,p) level of series **In**.

**Figure 5:**
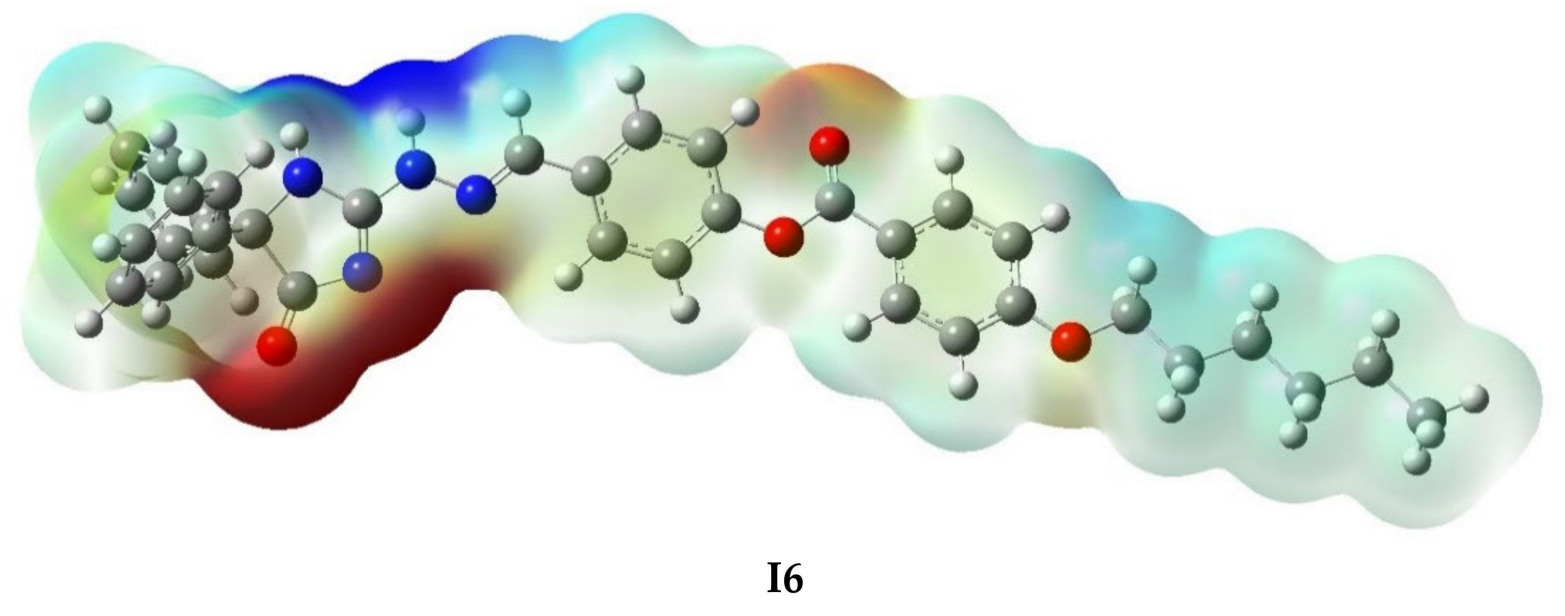

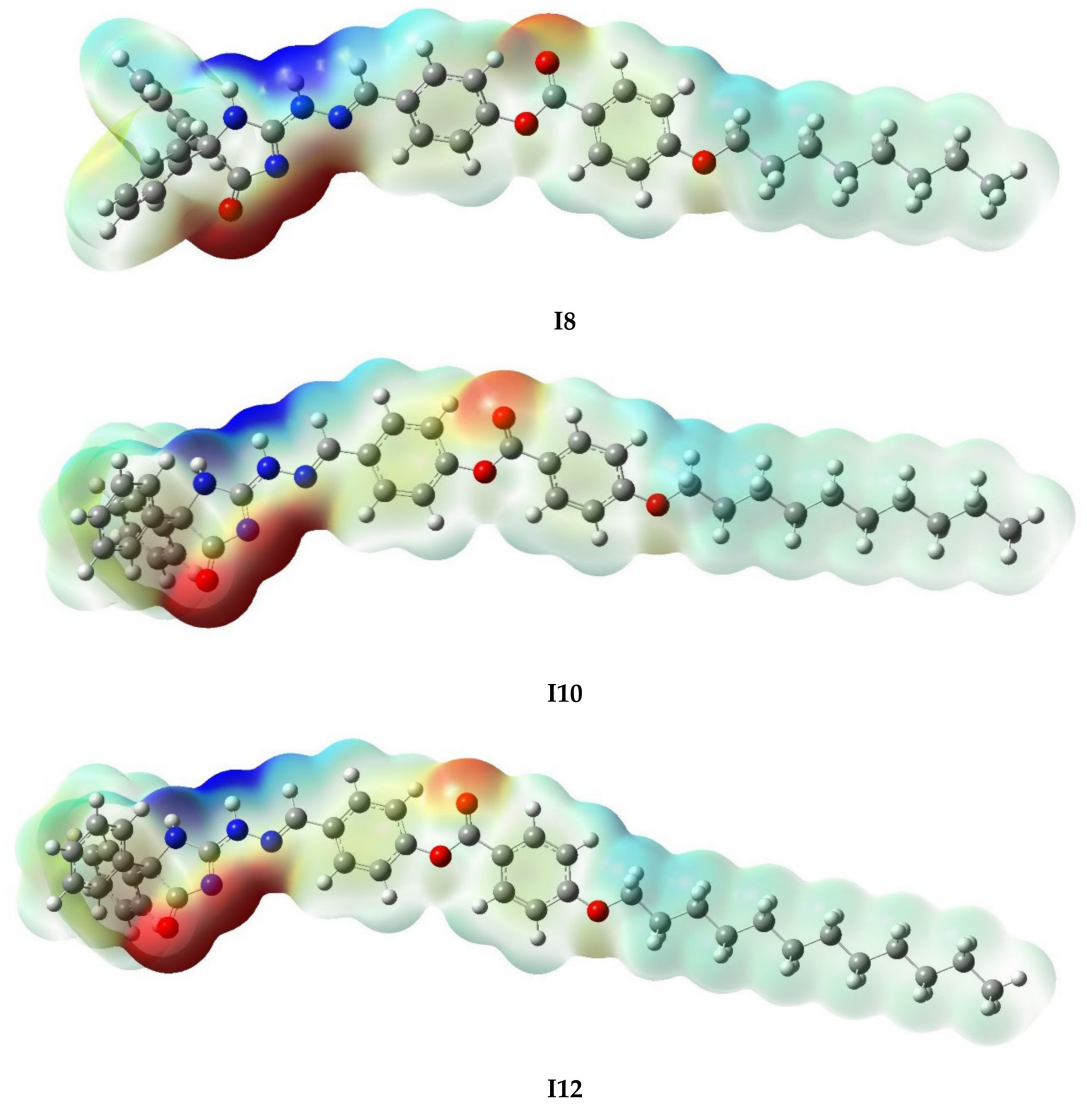
MEP computed for the B3LYP/6-31g(d,p) of series **In** at the electronic isosurface of 0.02 a.u.

The energy difference (E) between HOMO and LUMO levels, ionization potential (I.P), and electron affinity (EA) of chemical compounds are typically used to determine their reactivity [56,57]. As such, these parameters were calculated at B3LYP/6-31G (d,p) level and the results are presented in Table 2. Similar chemical reactivity was predicted for all the compounds (**In**) studied, as the values of parameters computed for each of the corresponding reactivity parameters were almost the same. This observation suggests that the reactivity of the derivatives is less sensitive to the size of the system [58]. On the part of the dipole moment highlighted in Table 2, the higher value calculated for **I10** over others is an indication of greater polarity. Moreover, the magnitude of the computed isotropic polarizability was found to be size dependent with the I12 having the largest value while the I6 has the least. Regarding the Frontier molecular orbitals (FMO’s) portrayed in Figure 4, the similar molecular distribution recorded for the corresponding HOMO and LUMO of all the derivatives could be attributed to the closeness in their respective HOMO and LUMO energy levels [57,58]. In the case of the HOMO, the electron clouds were steadily distributed over the nitrogen and oxygen atoms of imidazolone unit, nitrogen and carbon atoms together with π bond of the -HN-N=CH-linkage. This even electron cloud distribution extended to the carbon atoms and the π-electrons of the immediate phenyl ring to the -HN-N=CH- linkage as well as alkoxy oxygen of the –O-C=O linkage and the oxygen atom of the phenoxide ring. In addition, an appreciable electron density distribution was observed over some carbon atoms and π-electrons of the phenoxide. The high electron deficiency predicted for the terminal alkyl group could be associated with the consequential effect of the –O-C=O linkage that resonantly stabilizes the phenoxide ring via electron withdrawal [58]. On the part of the LUMO, the even spreading of electron clouds occurred over the nitrogen and oxygen atoms of imidazolone together with the sigma bond between the OC-N section of imidazole ring. This distribution got extended to nitrogen and carbon atoms together with π bond of the -HN-N=CH- linkage as well as the carbon atoms of the middle phenyl ring together with carbon and oxygen atoms of the carbonyl part of ester linkage. For the phenoxide section, the electron cloud distribution was found over only the ring carbon atoms and oxygen linking the terminal alkyl group. Concerning the molecular electrostatic potential (MEP) represented in Figure 5, the red cloud extending over the region of imidazolone oxygen to the -HN-N=CH- linkage indicates high electron density for this region but low electrostatic potential. Moreover, appreciable electron density was predicted over the carbonyl oxygen of the ester linkage. On the other hand, the blue shadow predicted for the region between the imidazole hydrogen and -HN-N=CH- linkage suggests a high electrostatic potential with low electron density.

Energy of a system is always size dependent because it is an extensive property of matter. This fundamental statement is justified by the calculated zero-point energy, thermal energy, and thermodynamic parameters showcased in Table 3 as their magnitude increases with the increasing size of the system. Moreover, the increasing magnitude of the calculated thermal energy and thermodynamic indicators with the size of the system is consistent with the observed trend in the smectogenic temperature range (**Δ*T***_SmC_) and thermal stability highlighted in Table 1. These experimental data are essential parameters in describing the mesomorphic characteristics.

## 3. Experimental

### 3.1. Synthesis

**Scheme 2:**
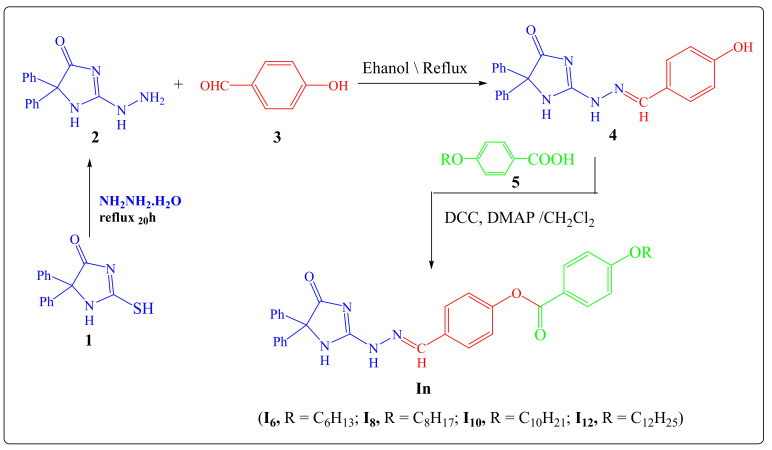
Synthesis of title series, **In**.

Compound (**2**) was synthesized following the procedures outlined in the literature [45] (see Supplementary Materials). The corresponding hydrazone derivative **4** was obtained by condensing compound **2** with 4-hydroxybenzaldehyde **3** in acetic acid containing a few drops of conc. hydrochloric acid (Scheme 2).

The isolated hydrazone derivative **4** mass spectra revealed molecular ion peaks at the predicted *m*/*z* value = 370. Their IR spectra revealed the absence of the NH_2_ group, as well as a carbonyl band at 1745 cm^−1^ and three bands at 3415, 3244, and 3194 cm^−1^ that were assigned to the OH and 2NH groups, respectively. The presence of azomethine and 2NH protons was also detected in ^1^H NMR spectra at = 8.19, 9.31, and 3.63 ppm, respectively.

Compound **4** was later converted to the respective **In**, via reaction with **5** in dry methylene chloride containing *N*,*N′−*dicyclohexylcarbodiimide (DCC) and catalytic amounts of 4*−*dimethylaminopyridine (DMAP) (Scheme 2).

### 3.2. Computational Details

Using the GAUSSIAN 09 software, the examined compounds were fully optimized without geometrical restrictions [46]. Then, using frequency calculation to confirm that all of the frequencies were actual, their global minimum optimization was ensured. In addition, after optimization, the Frontier molecular orbitals and the molecular electrostatic potential surfaces were produced from the check (chk) files. Density functional theory (DFT) and the B3LYP technique [47,48] were employed throughout the calculations, utilizing the basis set 6-31g (d,p) as a basis.

## 4. Conclusions

The novel imidazole liquid crystal homologues series, (*E*)-4-(2-(4-oxo-5,5-diphenyl-4,5-dihydro-1H-imidazol-2-yl)hydrazineylidene)methyl)phenyl4-(alkoxy)benzoate, was investigated experimentally and theoretically. The chemical structures of the produced materials were confirmed by FT-IR, NMR, and elemental analysis. DSC and POM were used to examine the mesomorphic properties of the homologues under investigation. All the derivatives were found to have smectogenic mesomorphic properties, as well as suitable SmC temperature ranges with enantiotropic features. Theoretical investigations revealed that increasing the length of the terminal group enhances the polarizability of the title compounds, while their HOMO–LUMO energy gap is less sensitive to the chain length. Other reactivity indictors suggested that the compounds reactivity was not significantly influenced by size of the system.

## Figures and Tables

**Table 1:** Phase transition temperatures upon heating and cooling rounds (°C), (enthalpy of transition **Δ*H***, kJ/mole), mesomorphic range (**Δ*T***), and normalized entropy of transition, **ΔS/R** for series **In**.

Comp.	*T*_Cr-SmC_	Δ*H*_Cr-SmC_	*T*_SmC-I_	Δ*H*_SmC_ *-*_I_	Δ*T*_SmC_	*T*_I-SmC_	Δ*H*_I-SmC_	*T*_SmC-Cr_	Δ*H*_SmC_ *-*_Cr_	Δ*S*_SmC-I_/R
**I6**	111.8	40.24	128.9	2.98	17.10	128.0	2.90	70.1	20.27	0.89
**I8**	107.3	38.55	131.1	3.05	23.80	130.0	2.85	68.9	18.55	0.91
**I10**	105.2	34.00	132.6	3.10	27.40	131.2	2.90	75.3	24.70	0.92
**I12**	90.8	45.20	133.5	2.93	42.70	132.1	1.97	80.2	25.29	0.87

Cr-SmC = transition from solid to the smectic C phase. SmC-I = transition from smectic C to the isotropic phase.

**Table 2:** Reactivity parameters, dipole moment and polarizability computed at B3LYP/6-31g(d,p) level.

Compound	E_HOMO_ (eV)	E_LUMO_ (eV)	∆E (eV)	Dipole Moment (Debye)	I.P (eV)	E.A (eV)	Isotropic Polarizability (Bohr**3)
**I6**	−5.972	−1.639	4.333	7.378	5.972	1.639	465.45
**I8**	−5.985	−1.625	4.360	7.482	5.985	1.625	487.34
**I10**	−5.978	−1.628	4.349	7.740	5.978	1.628	511.11
**I12**	−5.959	−1.642	4.317	7.507	5.959	1.642	535.72

**Table 3:** Zero-point energy, thermal energy and the thermodynamic parameters determined at B3LYP/6-31g(d,p) for group **In** at 298.15 K and 1 atm.

Compound	ZPE ^a^ (kcal/mol)	Thermal ^b^ (kcal/mol)	Enthalpy ^c^ (kcal/mol)	Gibbs’ Free Energy ^d^ (kcal/mol)	Entropy ^e^ (cal/mol K)
**I6**	392.8934237	417.4968357	418.0892052	341.3441047	257.405
**I8**	428.6395308	454.9491426	455.5421395	375.1261055	269.716
**I10**	464.4239161	492.4359625	493.0289594	408.2730662	284.273
**I12**	500.1687682	529.9215273	530.5138968	441.4896806	298.589

^a^$\;\sigma_{ZPE}$ = 39.98206, ^b^ $\;\sigma_{Thermal}\;$= 41.89954, ^c^ $\;\sigma_{Enthalpy}$= 41.89954, ^d^ $\;\sigma_{Gibbs^{\prime }free\;energy}$= 37.29614, ^e^ $\;\sigma_{Entropy}$= 15.45169.

## Data Availability

The data presented in this study are available on request from the corresponding author.

## Supplementary Materials

The following supporting information can be downloaded at: https://www.mdpi.com/article/10.3390/molecules27144607/s1.
