# Supplementary material for: Novel Imidazole Liquid Crystals; Experimental and Computational Approaches
Source: Molecules. 2022 Jul 19;27(14):4607. doi: 10.3390/molecules27144607 (PMC9316631; doi:10.3390/molecules27144607)
Supplement: Supplementary file 1 [file molecules-27-04607-s001.zip › molecules-1786286-supplementary.pdf]

Novel Imidazol liquid crystals; Experimental and computational approaches

Nada s. Al-Kadhi [1], Fowzia S. Alamro [1], Saheed A. Popoola [2], Sobhi M. Gomha [2,3,*], Noha S. Bedowr [4], Shahd S. Al-Juhani [4] and Hoda A. Ahmed [3,*]

[1]Department of Chemistry, College of Science, Princess Nourah bint Abdulrahman University, P.O. Box 84428, Riyadh 11671, Saudi Arabia; nsalkadhi@pnu.edu.sa (N.S.A.-K) ; fsalamro@pnu.edu.sa (F.S.A.)

[2]Chemistry Department, Faculty of Science, Islamic University of Madinah, Madinah 42351, Saudi Arabia; abiodun@iu.edu.sa

[3]Department of Chemistry, Faculty of Science, Cairo University, Cairo 12613, Egypt

[4]Chemistry Department, College of Sciences, Taibah University, Yanbu 30799, Saudi Arabia; nbedowr@taibahu.edu.sa (NS.B) ; tu4053324@taibahu.edu.sa (SS.A)

* Correspondence: smgomha@iu.edu.sa (S.M.G.); ahoda@sci.cu.edu.eg (H.A.A.)

1. *Materials*

Benzil, thiourea, 4-hydroxybenzaldehyde, 4-hexyloxybenzoic acid, 4-octyloxybenzoic acid, 4-decyloxybenzoic acid, 4-dodecyloxybenzoic acid were purchased from Sigma Aldrich (Germany). dichloromethane, while *N,N'*-dicyclohexylcarbodiimide (DCC), hydrazine hydrate, ethanol and 4-dimethylaminopyridine (DMAP) were purchased from Aldrich (Wisconsin, USA).

2. *Analyses of materials*

Synthesis of (E)-2-(2-(4-hydroxybenzylidene)hydrazineyl)-5,5-diphenyl-1,5-dihydro-4H-imidazol-4-one, 4

A mixture of 2-hydrazineyl-5,5-diphenyl-1,5-dihydro-4H-imidazol-4-one (2.66g, 10 mmol) and 4–hydroxybenzaldehyde (1.22g, 10 mmol) in ethanol (20 mL) were refluxed for two hours (monitored by TLC). The mixture was cooled to room temperature and filtered. The obtained solid was washed with cold ethanol and recrystallized twice from hot dioxane to give pure imine compound **3** as indicated by TLC analysis**:** White solid, yield: 85%; mp 232.3 $^{\circ}$C, FTIR (ύ, cm^{-1}): 3415 (OH), 3244, 3194 (2NH), 2922, 2849 (CH_2 stretching), 1745 (C=O), 1604 (C=N), 1563 (C=C), 1458 (C–O $_{Asym}$), 1253 (C-O $_{Sym}$); ^{1}H-NMR (400 MHz, $CDCl_3$): δ/ppm: 6.91-7.49 (m, 14H, Ar-H), 8.29 (s, 1H, CH=N), 8.63 (br s, 1H, NH), 9.31 (br s, 1H, NH), 10.75 (br s, 1H, OH); Ms m/z (%) 370 (M^+, 61). Anal. Calcd. for $C_{22}H_{18}N_4O_2$ (370.41): C, 71.34; H, 4.90; N, 15.13. Found: C, 71.16; H, 4.74; N, 15.01%.

General procedure for synthesis of (E)-4-((2-(4-oxo-5,5-diphenyl-4,5-dihydro-1*H*-imidazol-2-yl)hydrazineylidene)methyl)phenyl 4-(alkoxy)benzoate, In

A mixture of imine compound **4** (1.85g, 5 mmol) and the appropriate 4-alkoxy benzoic acid derivatives (5 mmol for each) in dry methylene chloride (20 mL) containing *N*, *N′*–dicyclohexylcarbodiimide (DCC, 5 mmol) and few crystals of 4–dimethylaminopyridine (DMAP), as catalyst, were left to react for 72 hours at room temperature with continuous stirring. The solid was then filtered off and the solution evaporated. The solid residue obtained was recrystallized from ethanol to give TLC pure products. The purity of the prepared samples was checked with thin-layer chromatography (TLC) using TLC sheets coated with silica gel (E Merck), and CH_2Cl_2/CH_3OH (9:1) as eluent, and only one spot was detected by a UV-lamp.

^{1}H-NMR and elemental analyses for the investigated compounds were consistent with the structures assigned. ^{1}H-NMR data showed the expected integrated aliphatic to aromatic proton ratios in all compounds investigated. The physical data of products **In** are listed below:

(E)-4-((2-(4-Oxo-5,5-diphenyl-4,5-dihydro-1*H*-imidazol-2-yl)hydrazineylidene) methyl)phenyl 4-(hexyloxy)benzoate I6:

Yield: 89.0%; mp 112.0 °C, ^{1}H-NMR (600 MHz, $CDCl_3$): δ/ppm: 0.75-0.83 (t, 3H, CH_3), 1.23-1.48 (m, 6H, $CH_3\underline{(CH_2)_3}CH_2CH_2O$-), 1.71-1.79 (m, 2H, $CH_3(CH_2)_3\underline{CH_2}CH_2O$-), 3.84-4.00 (t, 2H, $CH_3(CH_2)_3CH_2\underline{CH_2}O$-), 6.89-7.81 (m, 16H, Ar–H), 7.94-7.99 (d, 2H, Ar–H), 8.31 (s, 1H, CH=N), 9.19 (br s, 1H, NH), 10.05 (br s, 1H, NH); ^{13}C-NMR (600 MHz, $CDCl_3$): δ/ppm: 14.02 (CH_3), 21.38, 23.34, 27.60, 30.72, 68.47 (CH_2), 91.37, 116.03, 119.16, 120.25, 121.03, 121.64, 123.16, 130.72, 133.53, 141.59, 147.39, 150.39, 156.44, 162.48 (Ar-C and C=N), 166.08, 179.15 (2C=O). Anal. Calcd. for $C_{35}H_{34}N_4O_4$ (574.68): C, 73.15; H, 5.96; N, 9.75. Found: C, 73.03; H, 5.80; N, 9.59%.

(E)-4-((2-(4-Oxo-5,5-diphenyl-4,5-dihydro-1*H*-imidazol-2-yl)hydrazineylidene) methyl)phenyl 4-(octyloxy)benzoate I8: Yield: 88.0%; mp 107.0 °C, ^{1}H-NMR (600 MHz, $CDCl_3$): δ/ppm: 0.82-0.86 (t, 3H, CH_3), 1.22-1.46 (m, 10H, $CH_3\underline{(CH_2)_5}CH_2CH_2O$-), 1.70-1.76 (m, 2H, $CH_3(CH_2)_5\underline{CH_2}CH_2O$-), 3.87-4.03 (t, 2H, $CH_3(CH_2)_5CH_2\underline{CH_2}O$-), 6.87-7.91 (m, 16H, Ar–H), 7.96-8.05 (d, 2H, Ar–H), 8.30 (s, H, CH=N), 9.27 (br s, 1H, NH), 10.11 (br s, 1H, NH). Anal. Calcd. for $C_{37}H_{38}N_4O_4$ (602.74): C, 73.73; H, 6.36; N, 9.30. Found: C, 73.62; H, 6.52; N, 9.17%.

(E)-4-((2-(4-Oxo-5,5-diphenyl-4,5-dihydro-1*H*-imidazol-2-yl)hydrazineylidene) methyl)phenyl 4-(decyloxy)benzoate I10: Yield: 91.0%; mp 105.0 °C, ^{1}H-NMR (600 MHz, $CDCl_3$): *δ*/ppm: 0.81-0.83 (t, 3H, CH_3), 1.25-1.45 (m, 14H, $CH_3\underline{(CH_2)_7}CH_2CH_2O$-), 1.71-1.79 (m, 2H, $CH_3(CH_2)_7\underline{CH_2}CH_2O$-), 3.85-4.03 (t, 2H, $CH_3(CH_2)_7CH_2\underline{CH_2}O$-), 6.89-6.86 (m, 16H, Ar–H), 8.00-8.07 (d, 2H, Ar–H), 8.27 (s, 1H, CH=N), 9.21 (br s, 1H, NH), 10.07 (br s, 1H, NH); ^{13}C-NMR (600 MHz, $CDCl_3$): *δ*/ppm: 13.59 (CH_3), 21.03, 21.28, 23.37, 24.91, 27.00, 29.35, 30.47, 31.72, 68.47 (CH_2), 91.13, 116.06, 120.01, 120.41, 122.04, 122.70, 124.32, 129.48, 131.28, 139.93, 144.40, 151.15, 156.91, 162.96 (Ar-C and C=N), 166.05, 178.45 (2C=O). Anal. Calcd. for $C_{39}H_{42}N_4O_4$ (630.79): C, 74.26; H, 6.71; N, 8.88. Found: C, 74.39; H, 6.60; N, 8.65%.

(E)-4-((2-(4-Oxo-5,5-diphenyl-4,5-dihydro-1*H*-imidazol-2-yl)hydrazineylidene) methyl)phenyl 4-(dodecyloxy)benzoate I12:
Yield: 90.2%; mp 91.0 °C, ^{1}H-NMR (600 MHz, $CDCl_3$): *δ*/ppm: 0.78-0.84 (t, 3H, CH_3), 1.19-1.40 (m, 16H, $CH_3\underline{(CH_2)_9}CH_2CH_2O$-), 1.70-1.75 (m, 2H, $CH_3(CH_2)_9\underline{CH_2}CH_2O$-), 3.84-3.99 (t, 2H, $CH_3(CH_2)_9CH_2\underline{CH_2}O$-), 6.86-7.91 (m, 16H, Ar–H), 7.95-8.01 (d, 2H, Ar–H), 8.28 (s, 1H, CH=N), 9.15 (br s, 1H, NH), 10.017 (br s, 1H, NH). Anal. Calcd. for $C_{41}H_{46}N_4O_4$ (658.84): C, 74.74; H, 7.04; N, 8.50. Found: C, 74.63; H, 7.00; N, 8.37%.

3.Charactrizations

Melting points were determined by MEL-TEMP II melting point apparatus in open glass capillaries and presented uncorrected. The IR spectra were recorded using potassium bromide (KBr) discs on a Perkin-Elemer FT-IR (Fourier-Transform Infrared Spectroscopy), available at the college of Science, Taibah University. The NMR spectra were carried out at ambient temperature (~25 °C) on a (JEOL) 500 MHz spectrophotometer using tetra methyl silane (TMS) as an internal standard, at the NMR Unit, Faculty of Science, Mansoura University. Chemical shift was recorded as δ values in parts per million (ppm), and the signals were reported as s (singlet), d (doublet), t (triplet) and m (multiplet). Elemental analyses were analyzed at the Micro analytical Unit, Faculty of Science, Cairo University.

TA Instruments Co. (Q20 Differential Scanning Calorimeter, DSC; USA) was used for recording phase transitions. DSC calibration was carried out using lead and indium melting temperature and enthalpy. Samples of 2–3 mg were used in aluminum pans for DSC investigation. The heating rate was 10°C/min in nitrogen gas as an inert atmosphere (30 ml/min). All transitions measured were for the second heating scan.

Transition temperatures for the prepared compounds were checked and phases were identified by Polarized optical microscope (POM, Wild, Germany) attached with Mettler FP82HT hot stage.
